# Editorial: Recent advances in radiotheranostics

**DOI:** 10.3389/fnume.2024.1520778

**Published:** 2024-11-15

**Authors:** Chuangyan Zhai, Kondapa Naidu Bobba

**Affiliations:** ^1^ Department of Nuclear Medicine, Nanfang Hospital, Southern Medical University, Guangzhou, China; ^2^ School of Forensic Medicine, Southern Medical University, Guangzhou, China; ^3^ Departmen of Nuclear Engineering, University of Tennessee-Knoxville, Knoxville, TN, United States

**Keywords:** radiotheranostics, radioiodine, prostate cancer, neuroendocrine cancer, lutetium-177, radium-223

**Editorial on the Research Topic**
Recent advances in radiotheranostics

In recent years, radiotheranostics has rapidly evolved as a groundbreaking field that merges diagnostic and therapeutic capabilities to enhance precision medicine, particularly in oncology. By pairing radioactive isotopes with targeting molecules, radiopharmaceuticals enable precise disease visualization and selective therapeutic intervention, providing unique avenues for personalized treatment with minimized side effects. These advances are poised to redefine how clinicians approach complex diseases, most notably cancer, but increasingly extending into other fields such as cardiology and neurology. This Research Topic, *Recent Advances in Radiotheranostics*, brings together notable contributions in the development of novel radiopharmaceuticals, improvements in imaging techniques, and applications in clinical settings, offering fresh insights and practical advancements for researchers, clinicians, and industry leaders.

Radiotheranostics has gained prominence due to the integration of targeted therapeutic agents with advanced imaging technologies, which allow real-time disease tracking and personalized intervention. The first radiotheranostic, radioiodine-131 for thyroid cancer, was approved by the FDA in 1971. The field's growth has been fueled by developments such as PSMA-targeted therapies for prostate cancer, which improve both diagnosis and treatment outcomes, as well as radiolabeled somatostatin analogs for neuroendocrine tumors. Advances in preclinical and clinical evaluations have refined our understanding of how these agents function, leading to improved treatment protocols that optimize safety and efficacy. As this research topic demonstrates, radiotheranostics is increasingly viewed as a versatile tool for addressing a wide range of medical challenges, from cancer to cardiovascular conditions, and represents a significant step toward precision medicine.

This research topic honors Dr. Saul Hertz (1905–1950), a pioneer in the use of radioiodine for treating thyroid hyperthyroidism and establishing the clinical application of radionuclide therapy. Dr. Hertz served as the director of the Thyroid Clinic at Massachusetts General Hospital (MGH) from 1931 to 1943. In 1936, he posed the question: “Could iodine be made radioactive artificially?” This inquiry marked the beginning of his groundbreaking research on radioactive iodine.

In 1937, with physicist Dr. Arthur Roberts, Dr. Hertz conducted an animal study involving 48 rabbits, administering radioactive iodine-128. Their qualitative analysis showed that hyperplastic thyroid glands retained more radioactive iodine than normal glands, demonstrating the potential of radioactive iodine (RAI) as a tracer for thyroid physiology studies. Following this success, Dr. Hertz recognized that RAI could hold therapeutic promise for thyroid cancer. A $30,000 grant from New York's Markle Foundation facilitated the construction of a cyclotron at MIT, which produced iodine isotopes I-130 and I-131. On March 31, 1941, Dr. Hertz administered MIT-produced RAI to patient Elizabeth D at MGH, marking the first therapeutic use of radioiodine. This pioneering clinical application led to a series of trials, ultimately involving 29 patients. In 1942, Dr. Hertz reported his early clinical trial results to the Markle Foundation, further supporting RAI's efficacy in treating thyroid disease. This year commemorates the 75th anniversary of Dr. Hertz's first clinical use of RAI on January 1, 1941, at MGH for treating hyperthyroidism.

Sallam et al. present a bibliometric and scientometric analysis of PSMA-targeted radiotheranostics, offering a structured overview of research trends and identifying influential contributors within this expanding field. Using data spanning 30 years, the authors map the progression of PSMA-targeted diagnostics and therapeutics, drawing attention to emerging research themes and highlighting key researchers and institutions. This study provides a comprehensive knowledge framework for PSMA-based research, serving as a valuable resource for understanding the landscape of prostate cancer treatment and identifying promising future directions. As a foundational study within the field, it underscores the need for continuous knowledge exchange and collaboration to advance PSMA-targeted radiotheranostics.

Li et al. explore the diagnostic potential of a novel PET tracer, ^68^Ga-NOTA-MAL-Cys-MZHER2:342, for imaging HER2-positive lung adenocarcinoma. This tracer has demonstrated high specificity and stability in preclinical and clinical studies, positioning it as a valuable non-invasive tool for visualizing HER2 expression. By enabling clinicians to more accurately characterize HER2-positive tumors, this research contributes to more precise diagnostic and treatment planning for lung adenocarcinoma. This study's promising results suggest that such innovative tracers can broaden radiotheranostics application in oncology, where early and accurate diagnosis is crucial for improving treatment outcomes.

Anido-Herranz et al. investigate the outcomes and prognostic factors associated with radium-223 treatment in patients with metastatic castration-resistant prostate cancer. This observational study emphasizes the survival benefits of second-line radium-223 treatment, notably observing improved outcomes in patients who display a reduction in alkaline phosphatase levels, a key prognostic marker. This study's findings highlight that radium-223 has potential to enhance therapeutic strategies in advanced prostate cancer, reinforcing the importance of integrating radiotheranostics agents into treatment protocols for metastatic diseases. The work represents an important advance for radiotheranostics in metastatic cancer care, highlighting new opportunities to personalize therapy based on patient-specific biomarkers.

Iqbal et al. evaluate the long-term clinical outcomes of ^177^Lu-DOTATATE in patients with metastatic midgut neuroendocrine tumors, particularly in those receiving third-line treatment or higher, an area that has not been previously studied in patients with non-ileal primaries. This study demonstrates encouraging response rates, supporting the use of ^177^Lu-DOTATATE as an effective option for cases where other treatments may have been exhausted. By extending treatment options for patients with limited alternatives, this work underscores the impact of radiotheranostics in managing complex cases, advocating for its wider adoption in clinical practice for neuroendocrine tumors. This research not only reinforces the efficacy of ^177^Lu-DOTATATE but also underscores the potential of radiotheranostics to address the diverse needs of patients with advanced disease.

Yu et al. examine the diagnostic value of a multimodal spectral CT protocol developed for patients with atrial fibrillation undergoing radiofrequency ablation. By integrating delayed enhancement and iodine quantification, this protocol improves visualization of left atrial appendage thrombus, facilitating better preoperative assessment. The study further incorporates water-swallowing techniques, which enhance anatomical clarity, supporting precise imaging of the left atrium, pulmonary veins, and esophagus. This innovative approach expands the application of radiotheranostics beyond oncology, demonstrating its relevance to cardiology and enhancing diagnostic precision for complex cardiovascular procedures.

Collectively, the contributions to this research topic illustrate the breadth of radiotheranostics impact, from foundational knowledge and cutting-edge imaging agents to practical applications that benefit patients with challenging conditions. By advancing our understanding of how targeted radiopharmaceuticals function and expanding their use across diverse medical fields, these studies underscore radiotheranostics role in driving forward personalized medicine. As this field continues to evolve, further innovations in radiopharmaceutical development, imaging technologies, and clinical applications hold the promise of addressing increasingly complex diseases with a patient-centered approach.

